# 6-[4-Chloro-2-(trifluoro­meth­yl)phen­yl]-3-fluoro-2-methyl­pyridine

**DOI:** 10.1107/S1600536812046211

**Published:** 2012-11-17

**Authors:** S. Sreenivasa, K. E. Manojkumar, P. A. Suchetan, N. R. Mohan, B. S. Palakshamurthy, T. Srinivasan, D. Velmurgan

**Affiliations:** a Department of Studies and Research in Chemistry, Tumkur University, Tumkur, Karnataka 572 103, India; b Department of Studies and Research in Chemistry, U.C.S, Tumkur University, Tumkur, Karnataka 572 103, India; c Department of Studies and Research in Physics, U.C.S., Tumkur University, Tumkur, Karnataka 572 103, India; d Centre of Advanced Study in Crystallography and Biophysics, University of Madras Guindy Campus, Chennai 600 025, India

## Abstract

In the title compound, C_13_H_8_ClF_4_N, the dihedral angle between the benzene and pyridine rings is 59.8 (3)°. In the crystal, mol­ecules are stacked in columns along the *b* axis through weak C—H⋯π inter­actions.

## Related literature

For the biological activity of pyridine derivatives, see: Patrick & Kinsmar (1996 ▶); Hishmat *et al.* (1990 ▶); Doshi *et al.* (1999 ▶); Bhatt *et al.* (2001 ▶).
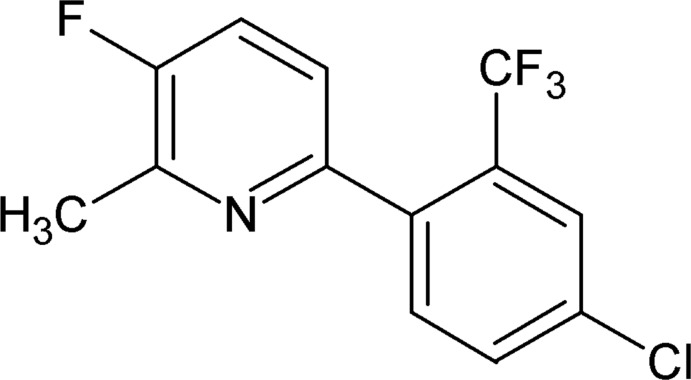


## Experimental

### 

#### Crystal data

C_13_H_8_ClF_4_N
*M* *_r_* = 289.65
Monoclinic, 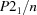

*a* = 13.1813 (7) Å
*b* = 4.5837 (3) Å
*c* = 20.4448 (11) Å
β = 92.441 (3)°
*V* = 1234.14 (12) Å^3^
*Z* = 4
Mo *K*α radiation
μ = 0.34 mm^−1^
*T* = 293 K
0.2 × 0.18 × 0.16 mm

#### Data collection

Bruker APEXII CCD area-detector diffractometer
11077 measured reflections
3033 independent reflections
2116 reflections with *I* > 2σ(*I*)
*R*_int_ = 0.031

#### Refinement

*R*[*F* ^2^ > 2σ(*F* ^2^)] = 0.042
*wR*(*F* ^2^) = 0.129
*S* = 1.01
3033 reflections
173 parameters
H-atom parameters constrained
Δρ_max_ = 0.23 e Å^−3^
Δρ_min_ = −0.30 e Å^−3^

### 

Data collection: *APEX2* (Bruker, 2004 ▶); cell refinement: *SAINT-Plus* (Bruker, 2004 ▶); data reduction: *SAINT-Plus*; program(s) used to solve structure: *SHELXS97* (Sheldrick, 2008 ▶); program(s) used to refine structure: *SHELXL97* (Sheldrick, 2008 ▶); molecular graphics: *ORTEP-3* (Farrugia, 2012 ▶); software used to prepare material for publication: *SHELXL97*.

## Supplementary Material

Crystal structure: contains datablock(s) global, I. DOI: 10.1107/S1600536812046211/is5209sup1.cif

Structure factors: contains datablock(s) I. DOI: 10.1107/S1600536812046211/is5209Isup2.hkl

Supplementary material file. DOI: 10.1107/S1600536812046211/is5209Isup3.cml

Additional supplementary materials:  crystallographic information; 3D view; checkCIF report

## Figures and Tables

**Table 1:** Hydrogen-bond geometry (Å, °) *Cg*1 is the centroid of the N1/C8–C12 ring.

*D*—H⋯*A*	*D*—H	H⋯*A*	*D*⋯*A*	*D*—H⋯*A*
C13—H13*B*⋯*Cg*1^i^	0.96	2.83	3.606 (2)	138

Symmetry code: (i) 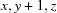.

**Fig. 1.**
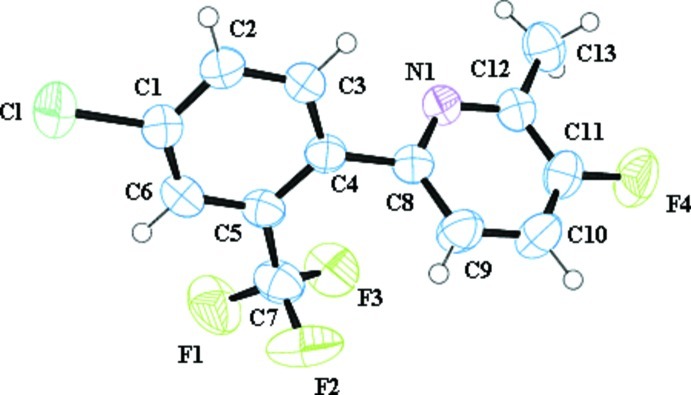
Molecular structure of the title compound, showing the atom-labeling scheme. Displacement ellipsoids are drawn at the 50% probability level.

**Fig. 2.**
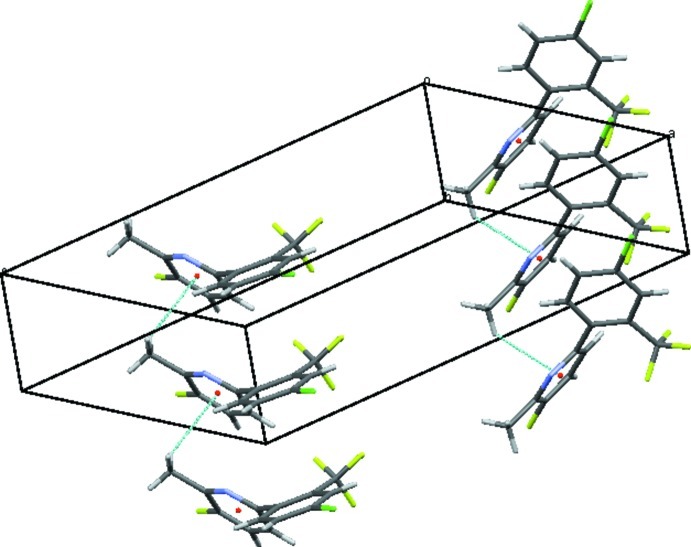
A packing diagram of the title compound, showing C—H··· π interactions (dotted lines).

## supplementary crystallographic information

### Comment

Pyridine derivatives of different heterocyclic nucleus have shown potent pharmacological properties like antifungal (Patrick & Kinsmar, 1996), antitubercular (Hishmat *et al.*, 1990) and antibacterial (Doshi *et al.*, 1999; Bhatt *et al.*, 2001). Keeping in view of the biological importance of this class of compound, we synthesized the title compound to study its X-ray crystal structure.

In the title compound, the dihedral angle between the least square planes through the benzene ring and the pyridine ring is 59.8 (3)°. In the absence of hydrogen bonds, the structure is stabilized by a weak intermolecular C—H···π interaction (Table 1).

### Experimental

2-Methyl-3-fluoropiridine-6-boromic acid (8.4 mmol) was taken in a mixture of dioxane (20 ml) and water (5 ml) at RT undernitrogen atmosphere. The reaction mixture was degassed with argon for 10 min, and K_2_CO_3_ (23.1 mmol) and dichlorobis(triphenylphosphene)-palladium(II) (0.231 mmol) were added and again degassed for 10 min. 2-Bromo-5-chlorobenzotrifluoride (7.7 mmol) was added and then reaction mixture was heated to 100 °C for 1 h (reaction was monitored by TLC), and the reaction mass was cooled to RT, diluted with ethyl acetate, filtered over celite, and washed with ethyl acetate. The filtrate was washed with water and brine, and the ethyl acetate layer was dried with anhydrous Na_2_SO_4_ and concentrated. The crude product was purified by tritulating with petroleum ether. Single crystals of the title compound used for X-ray diffraction studies were obtained from slow evaporation of the solution of the compound in petroleum ether-ethyl acetate mixture (1:1).

### Refinement

The H atoms were positioned with idealized geometry (C—H = 0.93–0.96 Å) using a riding model with *U*_iso_(H) = 1.2 or 1.5*U*_eq_(C).

### Crystal data

**Table:**

C_13_H_8_ClF_4_N	Prism
*M**_r_* = 289.65	*D*_x_ = 1.559 Mg m^−^^3^
Monoclinic, *P*2_1_/*n*	Melting point: 408 K
Hall symbol: -P 2yn	Mo *K*α radiation, λ = 0.71073 Å
*a* = 13.1813 (7) Å	Cell parameters from 2116 reflections
*b* = 4.5837 (3) Å	θ = 1.8–26.8°
*c* = 20.4448 (11) Å	µ = 0.34 mm^−^^1^
β = 92.441 (3)°	*T* = 293 K
*V* = 1234.14 (12) Å^3^	Prism, colourless
*Z* = 4	0.2 × 0.18 × 0.16 mm
*F*(000) = 584	

### Data collection

**Table:**

Bruker APEXII CCD area-detector diffractometer	2116 reflections with *I* > 2σ(*I*)
Radiation source: fine-focus sealed tube	*R*_int_ = 0.031
Graphite monochromator	θ_max_ = 28.3°, θ_min_ = 1.8°
Detector resolution: 1.20 pixels mm^-1^	*h* = −17→17
multi–scan	*k* = −5→6
11077 measured reflections	*l* = −27→27
3033 independent reflections	

### Refinement

**Table:**

Refinement on *F*^2^	Primary atom site location: structure-invariant direct methods
Least-squares matrix: full	Secondary atom site location: difference Fourier map
*R*[*F*^2^ > 2σ(*F*^2^)] = 0.042	Hydrogen site location: inferred from neighbouring sites
*wR*(*F*^2^) = 0.129	H-atom parameters constrained
*S* = 1.01	*w* = 1/[σ^2^(*F*_o_^2^) + (0.0549*P*)^2^ + 0.4806*P*] where *P* = (*F*_o_^2^ + 2*F*_c_^2^)/3
3033 reflections	(Δ/σ)_max_ = 0.001
173 parameters	Δρ_max_ = 0.23 e Å^−^^3^
0 restraints	Δρ_min_ = −0.30 e Å^−^^3^
0 constraints	

### Special details

**Table:**

Geometry. All e.s.d.'s (except the e.s.d. in the dihedral angle between two l.s. planes) are estimated using the full covariance matrix. The cell e.s.d.'s are taken into account individually in the estimation of e.s.d.'s in distances, angles and torsion angles; correlations between e.s.d.'s in cell parameters are only used when they are defined by crystal symmetry. An approximate (isotropic) treatment of cell e.s.d.'s is used for estimating e.s.d.'s involving l.s. planes.
Refinement. Refinement of *F*^2^ against ALL reflections. The weighted *R*-factor *wR* and goodness of fit *S* are based on *F*^2^, conventional *R*-factors *R* are based on *F*, with *F* set to zero for negative *F*^2^. The threshold expression of *F*^2^ > σ(*F*^2^) is used only for calculating *R*-factors(gt) *etc*. and is not relevant to the choice of reflections for refinement. *R*-factors based on *F*^2^ are statistically about twice as large as those based on *F*, and *R*- factors based on ALL data will be even larger.

### Fractional atomic coordinates and isotropic or equivalent isotropic displacement parameters (Å^2^)

**Table:**

	*x*	*y*	*z*	*U*_iso_*/*U*_eq_	
Cl1	0.83131 (5)	0.3848 (2)	0.03175 (3)	0.0883 (3)	
F1	1.12038 (13)	1.0835 (4)	0.09837 (7)	0.0978 (6)	
F2	1.18924 (9)	0.8102 (3)	0.17220 (10)	0.0964 (5)	
F3	1.11540 (11)	1.2001 (3)	0.19839 (7)	0.0781 (4)	
F4	1.09214 (11)	1.1818 (4)	0.46328 (6)	0.0853 (5)	
N1	0.93981 (11)	1.0496 (3)	0.31763 (7)	0.0451 (3)	
C1	0.87112 (13)	0.6135 (5)	0.21835 (9)	0.0528 (5)	
H1	0.8368	0.5883	0.2568	0.063*	
C2	0.83018 (14)	0.4976 (5)	0.16091 (9)	0.0589 (5)	
H2	0.7693	0.395	0.1605	0.071*	
C3	0.88083 (15)	0.5361 (5)	0.10418 (9)	0.0565 (5)	
C4	0.96922 (14)	0.6939 (5)	0.10389 (9)	0.0552 (5)	
H4	1.0015	0.7239	0.0649	0.066*	
C5	1.01029 (13)	0.8085 (4)	0.16185 (9)	0.0472 (4)	
C6	0.96206 (12)	0.7668 (4)	0.22062 (9)	0.0445 (4)	
C7	1.10812 (16)	0.9742 (5)	0.15823 (11)	0.0609 (5)	
C8	1.00181 (13)	0.8725 (4)	0.28603 (9)	0.0453 (4)	
C9	1.09464 (14)	0.7800 (5)	0.31331 (11)	0.0587 (5)	
H9	1.1349	0.6502	0.291	0.07*	
C10	1.12574 (16)	0.8841 (5)	0.37399 (11)	0.0638 (6)	
H10	1.1876	0.8282	0.3937	0.077*	
C11	1.06333 (16)	1.0706 (5)	0.40409 (10)	0.0571 (5)	
C12	0.96959 (14)	1.1523 (4)	0.37664 (9)	0.0485 (4)	
C13	0.90004 (18)	1.3525 (5)	0.41073 (10)	0.0642 (6)	
H13A	0.8421	1.3959	0.3823	0.096*	
H13B	0.9352	1.5301	0.422	0.096*	
H13C	0.8779	1.2605	0.4498	0.096*	

### Atomic displacement parameters (Å^2^)

**Table:**

	*U*^11^	*U*^22^	*U*^33^	*U*^12^	*U*^13^	*U*^23^
Cl1	0.0773 (4)	0.1319 (7)	0.0551 (3)	−0.0171 (4)	−0.0033 (3)	−0.0247 (4)
F1	0.1080 (12)	0.1124 (13)	0.0752 (9)	−0.0472 (10)	0.0308 (8)	0.0014 (9)
F2	0.0459 (7)	0.0725 (10)	0.1719 (16)	−0.0025 (6)	0.0166 (8)	−0.0082 (10)
F3	0.0911 (9)	0.0542 (8)	0.0897 (10)	−0.0197 (7)	0.0125 (7)	−0.0126 (7)
F4	0.0926 (10)	0.0965 (11)	0.0645 (8)	0.0001 (8)	−0.0251 (7)	−0.0226 (8)
N1	0.0467 (8)	0.0453 (8)	0.0433 (7)	0.0016 (6)	0.0034 (6)	0.0002 (6)
C1	0.0444 (9)	0.0704 (13)	0.0438 (9)	−0.0028 (9)	0.0057 (7)	0.0011 (9)
C2	0.0436 (9)	0.0797 (15)	0.0535 (11)	−0.0116 (10)	0.0012 (8)	−0.0005 (10)
C3	0.0505 (10)	0.0717 (14)	0.0471 (10)	0.0010 (10)	0.0000 (8)	−0.0079 (10)
C4	0.0539 (10)	0.0649 (13)	0.0477 (10)	0.0011 (9)	0.0122 (8)	−0.0030 (9)
C5	0.0432 (9)	0.0463 (10)	0.0526 (10)	0.0013 (8)	0.0078 (7)	−0.0038 (8)
C6	0.0410 (8)	0.0463 (10)	0.0464 (9)	0.0061 (7)	0.0032 (7)	−0.0021 (8)
C7	0.0592 (12)	0.0535 (12)	0.0710 (13)	−0.0077 (10)	0.0164 (10)	−0.0069 (11)
C8	0.0431 (9)	0.0460 (10)	0.0470 (9)	0.0014 (7)	0.0024 (7)	−0.0006 (8)
C9	0.0483 (10)	0.0615 (13)	0.0656 (12)	0.0120 (9)	−0.0042 (9)	−0.0081 (10)
C10	0.0507 (11)	0.0709 (15)	0.0684 (13)	0.0043 (10)	−0.0145 (9)	−0.0026 (11)
C11	0.0626 (12)	0.0567 (12)	0.0508 (10)	−0.0059 (10)	−0.0099 (9)	−0.0062 (9)
C12	0.0557 (10)	0.0437 (10)	0.0460 (9)	−0.0040 (8)	0.0030 (8)	0.0001 (8)
C13	0.0785 (14)	0.0619 (14)	0.0526 (11)	0.0043 (11)	0.0078 (10)	−0.0098 (10)

### Geometric parameters (Å, º)

**Table:**

Cl1—C3	1.737 (2)	C5—C7	1.501 (3)
F1—C7	1.339 (3)	C6—C1	1.388 (3)
F2—C7	1.328 (3)	C6—C5	1.396 (2)
F3—C7	1.322 (2)	C8—C9	1.389 (2)
F4—C11	1.353 (2)	C8—C6	1.496 (2)
N1—C8	1.338 (2)	C9—C10	1.375 (3)
N1—C12	1.338 (2)	C9—H9	0.93
C1—C2	1.378 (3)	C10—H10	0.93
C1—H1	0.93	C11—C10	1.352 (3)
C2—H2	0.93	C11—C12	1.387 (3)
C3—C2	1.374 (3)	C12—C13	1.491 (3)
C4—C3	1.372 (3)	C13—H13A	0.96
C4—H4	0.93	C13—H13B	0.96
C5—C4	1.385 (3)	C13—H13C	0.96
			
C8—N1—C12	119.16 (15)	F2—C7—C5	112.85 (18)
C2—C1—C6	121.99 (17)	F1—C7—C5	111.83 (18)
C2—C1—H1	119	N1—C8—C9	122.48 (17)
C6—C1—H1	119	N1—C8—C6	115.49 (15)
C3—C2—C1	118.89 (18)	C9—C8—C6	121.94 (17)
C3—C2—H2	120.6	C10—C9—C8	118.67 (19)
C1—C2—H2	120.6	C10—C9—H9	120.7
C4—C3—C2	120.98 (18)	C8—C9—H9	120.7
C4—C3—Cl1	119.69 (15)	C11—C10—C9	117.78 (18)
C2—C3—Cl1	119.33 (16)	C11—C10—H10	121.1
C3—C4—C5	119.83 (17)	C9—C10—H10	121.1
C3—C4—H4	120.1	F4—C11—C10	119.50 (18)
C5—C4—H4	120.1	F4—C11—C12	118.13 (19)
C4—C5—C6	120.57 (17)	C10—C11—C12	122.36 (18)
C4—C5—C7	117.07 (17)	N1—C12—C11	119.46 (18)
C6—C5—C7	122.37 (17)	N1—C12—C13	118.39 (17)
C1—C6—C5	117.69 (17)	C11—C12—C13	122.15 (18)
C1—C6—C8	117.57 (16)	C12—C13—H13A	109.5
C5—C6—C8	124.74 (16)	C12—C13—H13B	109.5
F3—C7—F2	105.85 (18)	H13A—C13—H13B	109.5
F3—C7—F1	105.45 (18)	C12—C13—H13C	109.5
F2—C7—F1	106.32 (18)	H13A—C13—H13C	109.5
F3—C7—C5	113.91 (17)	H13B—C13—H13C	109.5

### Hydrogen-bond geometry (Å, º)

*Cg*1 is the centroid of the N1/C8–C12 ring.

**Table:**

*D*—H···*A*	*D*—H	H···*A*	*D*···*A*	*D*—H···*A*
C13—H13*B*···*Cg*1^i^	0.96	2.83	3.606 (2)	138

Symmetry code: (i) *x*, *y*+1, *z*.
